# Electrospun Silver-Modified PZT/PVDF Composites for High-Performance Piezoelectric Energy Harvester

**DOI:** 10.3390/ma18071425

**Published:** 2025-03-24

**Authors:** Xiang Liu, Huiling Guo, Xinyue Yang, Fuling Wu, Yuanhui Li, Xiao Li, Qing Xu, Huajun Sun

**Affiliations:** 1 State Key Laboratory of Silicate Materials for Architectures, Wuhan University of Technology, Wuhan 430070, China; 331025@whut.edu.cn (X.L.); 13353695607@163.com (X.Y.); m15539774142@163.com (F.W.); 15093695638@163.com (Y.L.); 2 School of Materials Science and Engineering, Wuhan University of Technology, Wuhan 430070, China; 3 Advanced Ceramics Institute of Zibo New & High-Tech Industrial Development Zone, Zibo 255000, China; 4 College of Materials Science and Engineering, Hubei University of Automotive Technology, Shiyan 442002, China; 13837862582@163.com; 5 School of Chemistry, Chemical Engineering and Life Science, Wuhan University of Technology, Wuhan 430070, China; 18790187649@163.com

**Keywords:** piezoelectric composite, electrospinning, pPZT@Ag nanofibers, confined structure

## Abstract

Piezoelectric materials based on polyvinylidene fluoride (PVDF) are widely regarded as ideal candidates for the fabrication of piezoelectric energy harvesters (PEHs). However, the relatively low power output of PVDF limits its widespread application and poses a significant challenge to the advancement of PEHs. To address this issue, we have designed a novel PEH using silver-modified lead zirconate titanate/PVDF (pPZT@Ag/PVDF), which achieves a remarkable balance between high output performance and long-term stability. The pPZT@60Ag/PVDF PEH generates a peak voltage of 14.33 V, which is about 2.6 times that of the pure lead zirconate titanate/PVDF (pPZT/PVDF) PEH. This enhancement is attributed to the confined structure within the PVDF fibers, as well as the enhancement in dipole orientation alignment and the local electric field induced by silver nanoparticle modification. Furthermore, the pPZT@60Ag/PVDF PEH demonstrates a peak power density of 0.58 μW/cm^2^, with negligible degradation in output voltage after 6000 bending cycles, and efficiently harvests mechanical energy from human movement. This study presents an effective method for fabricating high-performance PEHs, which is expected to advance the development of next-generation energy harvesting devices.

## 1. Introduction

The escalating demand for wireless, portable, and self-powered devices, such as wearable technology, environmental sensors, and implantable medical devices, has stimulated considerable interest in flexible PEHs. These devices can effectively harvest energy from multiple sources, including environmental factors like wind and vibration, as well as mechanical energy produced by human movement [1,2,3]. The performance of PEHs is critically dependent on the piezoelectric materials employed. Traditional inorganic piezoelectric materials, such as lead zirconate titanate (PZT) [4,5], barium titanate (BTO) [6,7], and potassium sodium niobate (KNN) [8,9], showcase superior piezoelectric performance, but their inherent limitations, particularly high brittleness and low flexibility, constrain their application in self-powered flexible electronics. Among the various organic flexible piezoelectric materials, PVDF stands out due to its excellent electrical properties, flexibility, and mechanical properties [10,11,12,13]. There are three main phases, *α*, *β*, and *γ*, in PVDF [14]. The *α*-phase, belonging to the monoclinic crystalline system, lacks piezoelectric properties, while the *γ*-phase demonstrates a certain degree of piezoelectricity, although it is weaker than that of the *β*-phase. While pure PVDF has good flexibility, its inherent piezoelectric properties are limited, making it unable to meet escalating requirements.

To address the above challenges, the researchers investigated ways to integrate various piezoelectric ceramics into PVDF substrates. This approach is designed to combine the outstanding flexibility of PVDF with the excellent electrical properties of fillers. Although the piezoelectric filler can improve the output characteristics of the composite film, the highly resistive PVDF matrix consumes most of the applied polarization voltage, resulting in the effective electric field applied to the pPZT nanofibers being much lower than the theoretical value. In addition, the high resistance of the PVDF matrix reduces the charge transfer efficiency and suppresses the piezoelectric output to some extent. To tackle this problem, researchers have incorporated highly conductive fillers, such as graphene oxide (GO) [15,16], carbon nanotubes (CNTs) [17,18], and silver nanowires (Ag NWs) [19], into piezoelectric films. For instance, Wu et al. [15] showed that introducing graphene oxide into PZT/P(VDF-TrFE) films led to an outstanding open-circuit voltage of approximately 50 V with only 0.10 wt% doping. The addition of conductive fillers has indeed improved the piezoelectric output. However, challenges persist in achieving uniform dispersion.

Recently, the modification of conductive particles on the surface of piezoelectric fillers has become a new approach. Compared with the high surface energy of conductive fillers such as GO and CNTs, which are easily agglomerated in the PVDF matrix and isolated from piezoelectric fillers, loading conductive particles on the surface of piezoelectric fillers can make the conductive fillers uniformly distributed on the piezoelectric fillers, which improves the problem of easy agglomeration of the conductive fillers. The accumulated charge at the interface between the conductive filler and the PVDF matrix as well as the pPZT nanofibers also promotes the polarization of the piezoelectric filler and the PVDF matrix [20]. In addition, the conductive particles loaded on the surface of the piezoelectric filler can improve the piezoelectric charge transfer efficiency and thus increase the output voltage. For example, Zeng et al. [21] modified gallium metal particles onto PZT@GaOx fibers, which enhanced the polarization electric field and subsequently improved the output performance. This modification not only increased the content of the *β*-phase but also enhanced the polarizability of composite films. The PEH they fabricated has an open-circuit voltage (*V*_oc_) of 98.6 V, a short-circuit current (*I*_sc_) of 0.3 μA, and a power output of 9.8 μW. Similarly, Zhao et al. [22] employed a photoreduction method to modify silver nanoparticles onto BCZT. The resulting BCZT@Ag/PVDF composites exhibited an exceptional piezoelectric constant of 34 pC/N, representing a 2.6-fold increase compared to the BCZT/PVDF samples. As mentioned earlier, adding conductive nanoparticles to the surface of inorganic fillers is an effective strategy.

**Figure 1:**
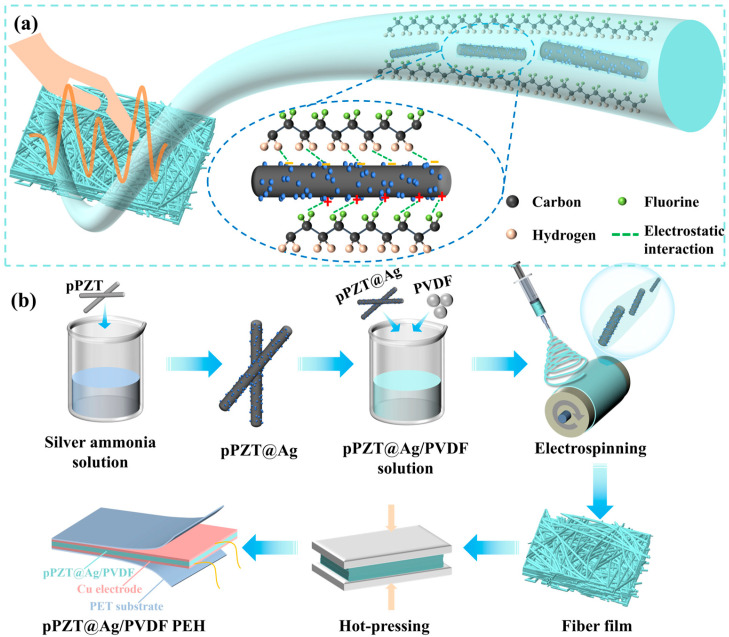
(**a**) A schematic diagram of the confined structure of the pPZT@Ag/PVDF fiber and (**b**) a schematic diagram of the preparation process of the pPZT@Ag/PVDF PEH.

This study explores the modification of PZT nanofibers with silver nanoparticles via a redox reaction, followed by embedding them into PVDF fibers through electrospinning. The problem of easy agglomeration of silver nanoparticles in the matrix can be improved by growing silver nanoparticles on the surface of PZT nanofibers uniformly in situ instead of doping them directly into the PVDF matrix. When fewer silver nanoparticles are loaded, the PVDF fibers maintain a confined structure (Figure 1a), and the formation of the *β*-phase and the polarization of the PVDF matrix are enhanced, resulting in a significant increase in piezoelectricity. The mean value of the piezoelectric constant of the pPZT@60Ag/PVDF composite fiber film was 25 pC/N, which was 122.5% higher than that of the pPZT/PVDF composite fiber film. This study presents an effective method for preparing composite fiber films with high-voltage electrical properties, which is expected to facilitate the development of flexible energy harvesters.

## 2. Materials and Methods

### 2.1. Materials

PVDF powder (Solvay 6010, Mw ≈ 600,000) was supplied by Solvay S.A., Brussels, Belgium. Tetrabutyl titanate (99.0%), zirconium acetylacetonate (98.0%), glacial acetic acid (99.5%), acetylacetone (99.0%), polyvinylpyrrolidone (Mw = 1,300,000), dopamine hydrochloride (98.0%), N,N-dimethylformamide (DMF, 99.5%), Tris-HCl buffer (pH = 8.5), silver nitrate solution (0.1 mol/L), and ammonium hydroxide (GR) were obtained from Aladin. Anhydrous ethanol (99.5%), basic lead acetate (99.5%), and acetone (99.5%) were supplied by China National Pharmaceutical Chemical Reagent Co. (Beijing, China).

### 2.2. Preparation of pPZT@Ag

In this experiment, polydopamine-coated PZT nanofibers (pPZT, as shown in Figure S1) were first prepared. Then, pPZT@Ag nanfibers were produced through the reduction of silver ions in silver–ammonia solution by phenolic hydroxyl groups on polydopamine. To prepare pPZT nanofibers with different silver nanoparticle loadings, 0.5 g of pPZT was mixed with 50 mL of silver–ammonia solution with different concentrations (x = 20, 40, 60, 80, or 100 mmol/L, where x is the concentration of silver–ammonia solution) and stirred for 0.5 h at 50 °C to allow for a full redox reaction. The reacted mixed solution was washed, centrifuged, and dried to obtain pPZT nanofibers with different silver nanoparticle loadings, defined as pPZT@xAg (x = 20, 40, 60, 80, or 100). The preparation process is illustrated in Figure 1b.

### 2.3. Preparation of pPZT@Ag/PVDF PEHs

The pPZT@Ag/PVDF precursor solution was prepared by dispersing 1.0 g of PVDF powder and 5 wt% pPZT@xAg into a mixed solvent of DMF and acetone (3:2), followed by stirring for 4 h at 60 °C. After ultrasonic dispersion, the solution was electrospun into pPZT@Ag/PVDF composite fiber films under an electric field of 1.5 kV/cm, a rotating speed of 2000 r/h, and an injection speed of 1 mL/h. To improve the self-supporting property and density of the composite fiber films, the three layers of composite fiber films (2.5 cm × 2.5 cm) were hot-pressed by a hot press for 0.5 h at a pressure of 10 MPa and a temperature of 25 °C to make them tightly bonded together. The hot-pressed composite fiber films were encapsulated to prepare pPZT@Ag/PVDF PEHs. Figure 1b shows a schematic diagram of the preparation process of pPZT@Ag/PVDF PEHs and pPZT/PVDF PEHs.

### 2.4. Characterization

X-ray powder diffraction (XRD, Bruker, D8 Advance, Billerica, MA, USA) patterns of pPZT and pPZT@Ag were recorded using Cu Kα radiation (λ = 1.5406 Å) (2θ of 10–70° in steps of 5°/min). The microstructure and elemental distribution of pPZT@Ag were examined using a high-resolution transmission electron microscope (HRTEM, Talos F200S, Thermo Fisher Scientific, Waltham, MA, USA) at an accelerating voltage of 120 kV, equipped with an EDAX Elite T spectrometer for compositional analysis. The surface morphology of pPZT@Ag and pPZT fibers, as well as pPZT/PVDF and pPZT@Ag/PVDF composite fiber films, was examined by scanning electron microscopy (SEM, Apreo 2S, Thermo Fisher Scientific, Waltham, MA, USA) at 15 kV. The elemental composition and binding states of pPZT@Ag surfaces were explored using X-ray photoelectron spectroscopy (XPS, AXIS SUPRA+, Cortos Analytical Instruments, Inc., Kyoto, Japan) with Al Kα radiation (1486.6 eV). Additionally, Fourier transform infrared (FTIR, NicoletS50, Thermo Fisher Scientific, Waltham, MA, USA) measurements of the polar-phase content in the fiber membrane were conducted. A differential scanning calorimeter (DSC8500, TA Instruments, Waltham, MA, USA) was used to record crystallinity. Bending of the PEH was achieved using a homemade pressure system (Figure S2). The piezoelectricity of pPZT/PVDF and pPZT@Ag/PVDF composite fiber films was measured using a quasi-static tester (ZJ-4AN, Institute of Acoustics, Chinese Academy of Sciences, Beijing, China). The dielectric properties of the composite fiber membranes were characterized from 10 Hz to 1 MHz using a broadband dielectric spectrometer (Concept 80, Novocontrol GmbH, Montabaur, Germany) under 1 V AC bias. A heat press (YLJ-100E, Hefei Kejing Material Technology Co., Hefei, China) was used to press the three layers of composite fiber film together to increase the self-support and density of the composite fiber film. We evaluated PEH output performance with an electrometer (Keithley 6514, Keithley Instruments, Cleveland, OH, USA).

## 3. Results and Discussion

The XRD patterns of pPZT and pPZT@Ag are shown in Figure S3. The diffraction peaks of all samples are in correspondence with the (100), (101), (111), (200), (201), (211), and (022) crystal planes in standard PDF (PDF# 057-0525). This suggests that the samples prepared in this study possess good crystallinity and a perovskite structure [23]. In addition, the XRD peaks of silver (2θ values of 38.2°, 44.4°, and 64.6°) overlapped significantly with the characteristic diffraction peaks of pPZT. Consequently, no distinct silver diffraction peaks can be observed in the XRD patterns of pPZT@Ag.

**Figure 2:**
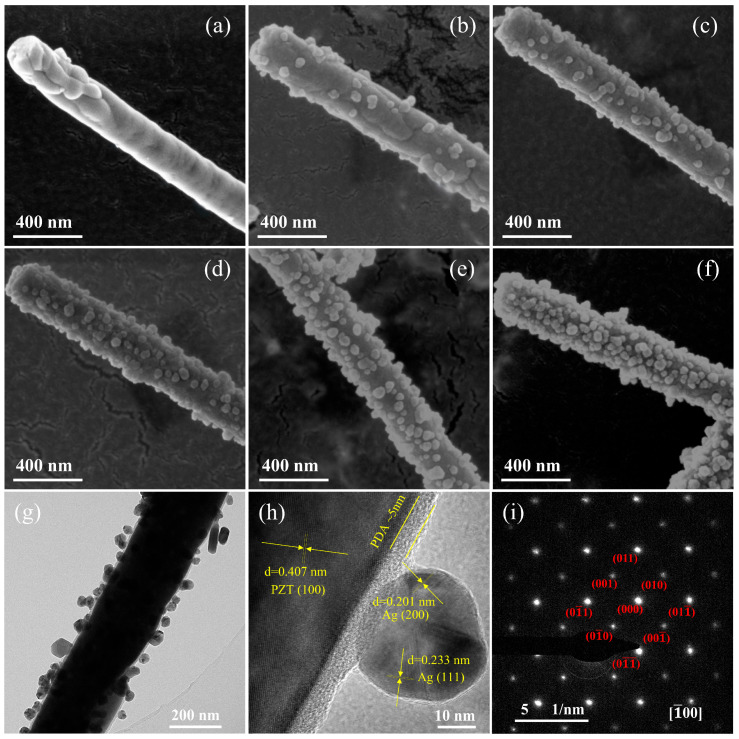
SEM images of (**a**) pPZT, (**b**) pPZT@20Ag, (**c**) pPZT@40Ag, (**d**) pPZT@60Ag, (**e**) pPZT@80Ag, and (**f**) pPZT@100Ag. (**g**) TEM image and (**h**) HRTEM image of pPZT@60Ag. (**i**) Selected Area Electron Diffraction of pPZT.

The surface morphology of pPZT and pPZT@Ag was inspected through SEM. As presented in Figure 2a, the surface morphology of pPZT is smooth and dense. On the contrary, Figure 2b–f reveals that the pPZT@Ag surface is loaded with nanoparticles, with their loading content increasing as the concentration of the silver–ammonia solution used in the synthesis process rises. To affirm the characteristics of these nanoparticles and observe the morphology and elemental distribution of pPZT@Ag on a more microscopic scale, HRTEM and EDS were employed. HRTEM images of pPZT@60Ag surface nanoparticles show two non-parallel lattice fringes with the spacing of 0.233 nm and 0.201 nm belonging to the (111) and (002) crystal planes of cubic silver, respectively [20], which confirms that the nanoparticles are silver. In addition, the 0.407 nm facet corresponds to the (100) facet in the XRD pattern of pPZT [21]. It can also be discerned from the TEM images that the surface of pPZT@60Ag is encapsulated with an amorphous polydopamine layer approximately 5 nm thick. Figure 2i shows the Selected Area Electron Diffraction map of pPZT, and in Figure 2h, the calibration of the diffraction spots identifies pPZT as a cubic perovskite structure. Figures S4 and S5 show the EDS plots of pPZT@60Ag and the content of each element, where silver (Ag) is distributed in granular form on the surface of pPZT, while lead (Pb), zirconium (Zr), titanium (Ti), oxygen (O), and carbon (C) are uniformly distributed throughout the sample.

**Figure 3:**
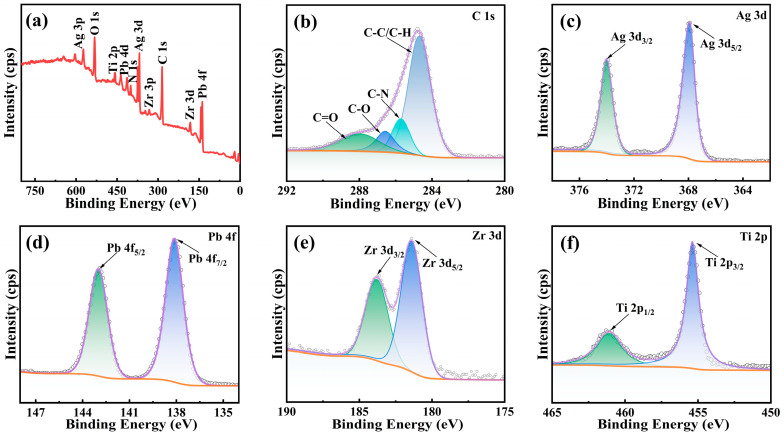
The full spectrum of the XPS (**a**) for pPZT@60Ag and the fine spectrum of (**b**) C1s, (**c**) Ag3d, (**d**) Pb4f, (**e**) Zr3d, and (**f**) Ti2p.

Figure 3a displays the full spectrum of the XPS for pPZT@60Ag after calibration, as well as fine spectra (Figure 3b–f) for C, Ag, Pb, Zr, and Ti. As shown in Figure 3b, the C1s spectrum was fitted to four peaks, C–C/C–H (284.66 eV), C–N (285.71 eV), C–O (286.57 eV), and C=O (287.94 eV), due to the presence of surface dopamine. The 3d orbital spectrum of Ag in Figure 3c reveals two peaks located at 368.19 eV (Ag3d_5/2_) and 374.30 eV (Ag3d_3/2_) [24], which suggests that the silver nanoparticles were successfully modified onto the pPZT surface. The 4f orbital spectrum of Pb splits into two peaks at 142.58 eV (4f_5/2_) and 137.79 eV (4f_7/2_). Similarly, the 2p orbital spectrum of Ti shows two peaks located at 463.95 eV (2p_1/2_) and 458.15 eV (2p_3/2_), while the 3d orbital spectrum of Zr shows two peaks located at 183.08 eV (3d_3/2_) and 181.13 eV (3d_5/2_). These results confirm that pPZT@Ag synthesized in this study has the correct elemental composition and a stable valence state.

**Figure 4:**
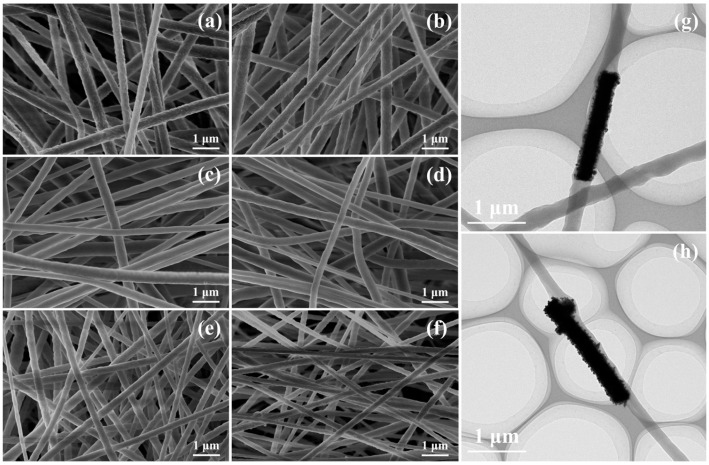
SEM images of (**a**) pPZT/PVDF composite fiber film and pPZT@Ag/PVDF composite fiber films with different silver nanoparticle loadings: (**b**) pPZT@20Ag, (**c**) pPZT@40Ag, (**d**) pPZT@60Ag, (**e**) pPZT@80Ag, and (**f**) pPZT@100Ag. (**g**) TEM of distribution state of pPZT@Ag in coarse PVDF fibers and (**h**) fine PVDF fibers.

Figure 4a–f present the surface morphology of the pPZT/PVDF composite fiber film and pPZT@Ag/PVDF composite fiber film. The fibers are cylindrical and the diameters follow a normal distribution, indicating that the spinning parameters employed in this study are well optimized. The statistical distribution of the pPZT/PVDF fiber diameter and the pPZT@Ag/PVDF fiber diameter is shown in Figure S6, with average diameters ranging from 211 to 370 nm. Notably, the diameter of the fibers decreases as the loading content of silver nanoparticles on pPZT increases. This phenomenon can be ascribed to the increased loading of silver nanoparticles, which enhances the electrical conductivity of the spinning solution. As a result, the solution jet experiences a stronger electric field during the electrospinning process, leading to further stretching of the PVDF fibers and a reduction in diameter [25]. Figure 4g and h further illustrate the distribution state of pPZT@Ag within the coarse and fine PVDF fibers. The coarse PVDF fibers can fully encapsulate pPZT@Ag, resulting in a confined structure. In contrast, the fine PVDF fibers do not completely enclose the pPZT@Ag, leaving parts of it exposed and forming a nonconfined structure [26].

**Figure 5:**
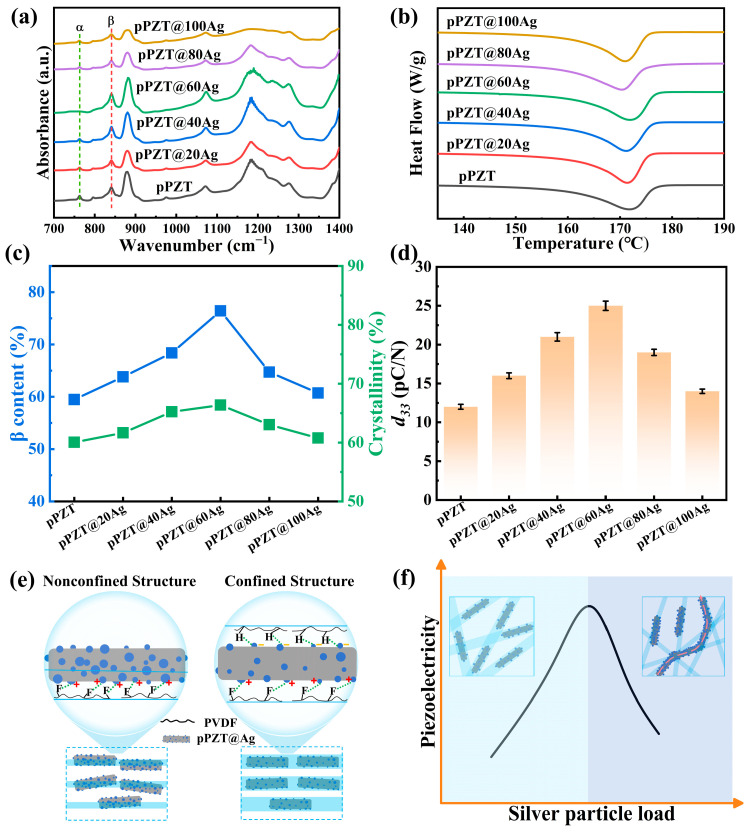
(**a**) The FTIR spectra and (**b**) DSC analysis of pPZT/PVDF and pPZT@Ag/PVDF composite fiber films. The (**c**) *β*-phase content and crystallinity test results, and (**d**) the average values of the *d*_33_ test results for the pPZT/PVDF and pPZT@Ag/PVDF composite fiber films. (**e**) A schematic of the interaction mechanism between pPZT@Ag and PVDF as well as a schematic of the closed and unconfined structures. (**f**) A schematic diagram of the percolation path in a composite fiber film.

The FTIR and DSC curves of pPZT/PVDF and pPZT@Ag/PVDF composite fiber films are shown in Figure 5a,b, respectively. The *β*-phase content can be calculated from Equations (1) and (2) [27,28,29,30,31].

(1) $$F_{EA}=A_{EA}/(A_{EA}+1.26A_{\alpha })$$

(2) $$F_{\beta }=F_{EA}\times \left(\frac{{∆H}_{\beta }}{{∆H}_{\beta }+{∆H}_{\gamma }}\right)$$

where *F_EA_* represents the relative fraction of the electroactive phases, while *A_EA_* and *A_α_* represent the absorbance peaks at 840 cm^−1^ and 761 cm^−1^. *F_β_* is the net content of the *β* phase in the composite fiber films. Δ*H_β_* and Δ*H_γ_* are the height differences (absorbance differences) between the peak around 1275 cm^−1^ and the nearest valley around 1260 cm^−1^, and the peak around 1234 cm^−1^ and the nearest valley around 1225 cm^−1^, respectively. As depicted in Figure 5c, the *β*-phase content in the composite fiber films initially increases with the increase in silver nanoparticle loading on the pPZT surface, ascending from 59.47% in pPZT/PVDF to 76.42% in pPZT@60Ag/PVDF. Nevertheless, when the silver nanoparticles are overloaded (x > 60), the *β*-phase content begins to decrease. This observed tendency can be ascribed to two crucial factors. Firstly, the existence of silver nanoparticles enhances the conductivity of the spinning solution, enabling the PVDF fibers to undergo a stronger electric field during the electrospinning process. This enhanced electric field significantly promotes the generation of the *β*-phase [32]. In addition, compared to pPZT, the pPZT@Ag fibers are subjected to a more intense local electric field, which enhances their polarization. This leads to the generation of induced charges on the pPZT@Ag surface which attract the -CF_2_ and -CH_2_ groups in the PVDF molecules, helping to align them and promoting the generation of the *β*-phase [33,34,35] (Figure 5e). However, when the loading of silver nanoparticles surpasses a certain threshold (x > 60), the electrical conductivity of the spinning solution increases significantly, resulting in a sharp reduction in the diameter of the PVDF fibers and the generation of breakdowns. An unconfined structure is formed in the fine fibers (Figure 5e), exposing more pPZT@Ag on the fiber surface, which inhibits *β*-phase formation. Simultaneously, the breakdown of the fibers increases the leakage current, leading to the insufficient polarization of PVDF.

Figure 5b shows the DSC curves of pPZT/PVDF and pPZT@Ag/PVDF composite fiber films. The crystallinity can be calculated using Equation (3) [36,37,38].

(3) $$X_{C}={\Delta H}_{m}/{\Delta H}_{100}$$

where Δ*H_m_* is the enthalpy of melting of the sample obtained from the test, and Δ*H*_100_ is the enthalpy of melting of the theoretically 100% crystallized sample (Δ*H*_100_ = 103.40 J/g). The crystallinity of the composite fiber films follows a trend similar to that of the *β*-phase content. Specifically, the crystallinity for the pPZT/PVDF and pPZT@xAg/PVDF composite fiber films (x = 20, 40, 60, 80, and 100) is measured at 60.04%, 61.65%, 65.22%, 66.37%, 63.03%, and 60.76%, respectively. Notably, the crystallinity of the pPZT@60Ag/PVDF composite fiber films shows the most remarkable increase, rising by 10.5% compared to pPZT/PVDF. However, when the loading of silver nanoparticles on pPZT exceeds a certain threshold (x > 60), a decrease in crystallinity is observed. This phenomenon can be explained by the fact that when the silver nanoparticle loading is low (x < 60), the PVDF fibers have a larger diameter, allowing the pPZT@Ag to be fully encapsulated within the fibers. This maximizes their role as nucleating agents, as shown in Figure 5e. However, as the silver nanoparticle content increases beyond this point (x > 60), the diameter of the PVDF fibers decreases. As depicted in Figure 5e, the distribution of pPZT@Ag in the fine PVDF fibers leads to a nonconfined structure. This weakens the role of pPZT@Ag as a nucleating agent, ultimately causing a reduction in the overall crystallinity of the composite fiber membranes.

The piezoelectric constant (*d*_33_) is a key indicator of piezoelectric properties, making the measurement of *d*_33_ essential for evaluating performance. To guarantee accuracy, ten tests were carried out, and the average value was regarded as the final *d*_33_. As presented in Figure 5d, the *d*_33_ values of the composite fiber films ascend with the augmentation in the loading content of silver nanoparticles on pPZT. Notably, the mean value of then piezoelectric constant of then pPZT@60Ag/PVDF composite fiber film is 25 pC/N, which is a 112.5% improvement compared to the 12 pC/N of pPZT/PVDF. Nevertheless, as the loading content of silver nanoparticles increases (x > 60), a noticeable decline in *d*_33_ is observed. This is because when the silver nanoparticle load content is low (x < 60), the spun PVDF fibers have a thicker diameter, facilitating complete encapsulation of pPZT@Ag and thereby establishing a confined structure. In this configuration, pPZT@Ag intensifies the local electric field, promoting polarization in PVDF and the arrangement of the -CF_2_ and -CH_2_ groups. Conversely, with the growth in silver nanoparticle loading (x > 60), the diameter of the PVDF fibers diminishes, preventing full encapsulation of pPZT@Ag and leading to a nonconfined structure. This nonconfined configuration leads to aggregation and direct contact among pPZT@Ag, creating percolation paths that suppress the piezoelectric properties of the composite film (a schematic illustration of the percolation paths is shown in Figure 5f) [26].

**Figure 6:**
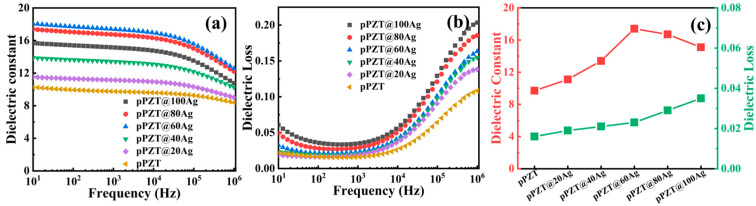
Frequency dependence of (**a**) *ε_r_* and (**b**) tanδ for pPZT/PVDF and pPZT@Ag/PVDF composite fiber films and (**c**) *ε_r_* and tanδ at 10^3^ Hz.

Figure 6a,b depict the dielectric constant (*ε_r_*) and dielectric loss (tanδ) as functions of electric field frequency for pPZT/PVDF and pPZT@Ag/PVDF fiber films. As illustrated in Figure 6a, the *ε_r_* of the fiber membrane is inversely correlated with the test frequency. This is because at higher frequencies the electric field transitions faster than at the relaxation frequency of the material, hindering the migration of polar groups and leading to dielectric relaxation phenomena [39]. Moreover, the *ε_r_* of the composite fiber membranes initially rises and then drops as the loading of silver nanoparticles on pPZT increases. The initial enhancement is attributed to the formation of numerous interfaces between pPZT and the silver nanoparticles upon their deposition. These interfaces promote the accumulation of space charge [9], enhancing interfacial polarization and thereby increasing the *ε_r_*. However, when more silver nanoparticles are loaded (x > 60), the exposed pPZT@Ag on the outer surface of the PVDF fibers tends to interact, neutralizing the induced charge, as depicted in Figure 5f. This interaction results in a decrease in the *ε_r_*. At a test frequency of 10^3^ Hz, the *ε_r_* measured for the pPZT/PVDF and pPZT@xAg/PVDF composite fiber films with varying silver nanoparticle loadings (x = 20, 40, 60, 80, and 100) is 9.7, 11.1, 13.4, 17.4, 16.7, and 15.1, respectively.

As shown in Figure 6b, the tanδ of pPZT/PVDF and pPZT@Ag/PVDF fiber films first decreases and then increases with increasing frequency. This phenomenon can be attributed to the combined effect of conductivity loss and polarization loss in PVDF [40]. At low frequencies (<10^3^ Hz), the polarization of the composite film can respond adequately to changes in the electric field, resulting in negligible polarization loss. Consequently, the conductive losses dominate. In contrast, at high frequencies (>10^3^ Hz), the dipole polarization loss of the matrix increases while the conductive loss decreases. The increase in polarization loss outweighs the decrease in conductive loss, leading to an increase in tanδ [41]. It is noteworthy that at a frequency of 10^3^ Hz, the tanδ of the fiber membrane is positively correlated with silver nanoparticle loading, where the tanδ of the pPZT@100Ag/PVDF fiber film is measured to be 0.035, which is about 2.2 times that of pPZT/PVDF (0.016). In addition, to integrate the dielectric constant and *d*_33_ value of the piezoelectric composite fiber membrane, the piezoelectric performance of the piezoelectric energy harvester can be described by the quality factor (FoM). The quality factor FoM can be calculated according to Equation (4) [42,43].

(4) $$FoM=\frac{d_{33}\times d_{33}}{\varepsilon_{o}\times \varepsilon_{r}}$$

where *d*_33_ denotes the piezoelectric coefficient, *ε*_0_ denotes the vacuum permittivity, *ε_r_* denotes the relative permittivity at a frequency of 10^3^ Hz, and *FoM* represents the quality factor. As can be seen from Figure S7, similar to the trend of *d*_33_ of the piezoelectric composite fiber membrane, the *FoM* value increases and then decreases with the increase in silver nanoparticle loading on pPZT. This indicates that the pPZT@60Ag/PVDF composite piezoelectric fiber membrane has the best piezoelectric performance.

**Figure 7:**
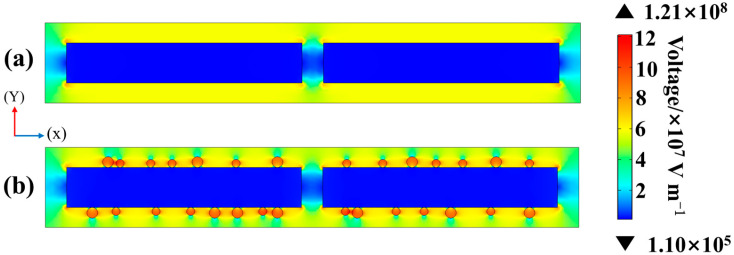
Simulated electric field distribution of (**a**) pPZT/PVDF fiber and (**b**) pPZT@60Ag/PVDF fiber.

The simulation results are shown in Figure 7a,b. To investigate the effect of silver nanoparticle loading on the polarization process of PVDF fibers, the electric field in PVDF fibers was simulated using COMSOL Multiphysics 5.6. The electrostatic physical field interface was chosen for the simulation, Gauss’s law was used for the equations, the finite element model was a Lagrangian-type cell, and the simulation process was carried out under steady-state conditions. In the established two-dimensional (2D) model, the outer and inner rectangles represent PVDF fibers and pPZT with lengths (*X*-axis) and widths (*Y*-axis) of 2 × 0.3 μm and 0.88 × 0.15 μm, respectively, and the circles represent silver nanoparticles with diameters of 0.03 and 0.04 μm. The dielectric constants of the PZT nanofibers, PVDF nanofibers, and silver nanoparticles were set to 504, 12, and 1, respectively, for the simulations. The bottom edge of the PVDF model was fixed and grounded, and an electric field of 30 kV/mm was applied along the *Y*-axis at the upper boundary of the model. The maximum electric field in pPZT/PVDF fibers is 7.74 × 10^7^ V/m. By contrast, the maximum electric field strength of pPZT@60Ag/PVDF fibers is 1.21 × 10^8^ V/m, which increased by 56.3% compared to pPZT/PVDF fibers. This increased local electric field exerts a torque on the dipole, forcing the molecular chains to orient along the direction of the electric field, and this orientation inhibits the formation of the nonpolar *α*-phase and promotes the nucleation and growth of the *β*-phase. The enhancement in the local electric field also accelerates the mobility of the molecular chains and shortens the induction time of *β*-phase nucleation. The increased local electric field promotes the PVDF fiber membrane to obtain a higher *β*-phase content through the above mechanism.

**Figure 8:**
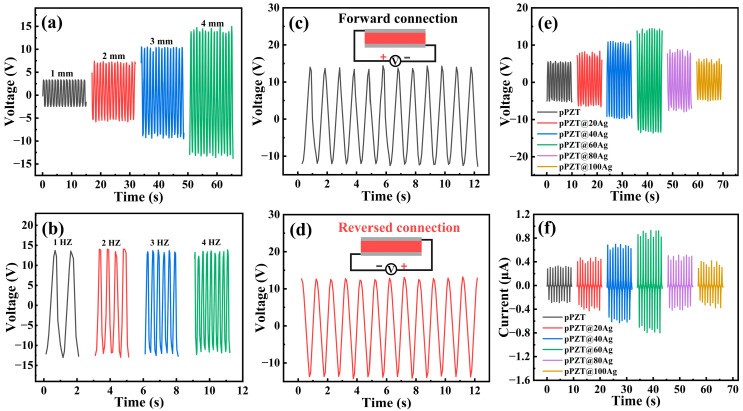
*V*_oc_ of pPZT@60Ag/PVDF PEH at (**a**) 1 Hz with different amplitudes and (**b**) 4 mm with different frequencies. (**c**) Forward and (**d**) reverse output voltage tests. (**e**) *V*_oc_ and (**f**) *I*_sc_ of pPZT/PVDF PEH and pPZT@Ag/PVDF PEHs.

To assess the piezoelectric output performance, the composite fiber films were encapsulated into PEHs, as presented in Figure 1b. Since both the test frequency and bending amplitude have a significant impact on the electrical output, we concentrated on evaluating the electrical output of the pPZT@60Ag/PVDF PEH under various conditions to determine the optimal configuration. As illustrated in Figure 8a, at a frequency of 1 Hz, the *V*_oc_ of the pPZT@60Ag/PVDF PEH increases with bending amplitude, reaching a peak when the bending amplitude arrives at 4 mm. This enhancement can be ascribed to the augmented effective strain and strain rate in the PVDF matrix at larger bending displacements, which facilitates greater polarization within the fiber film [44]. In contrast, as shown in Figure 8b, the influence of the test frequency on the *V*_oc_ is negligible, revealing only minor variations in the pPZT@60Ag/PVDF PEH across bending amplitudes of 4 mm and test frequencies ranging from 1 to 4 Hz. Hence, to optimize output performance, we chose 1 Hz and 4 mm as subsequent test conditions.

Forward and reverse connection experiments were conducted before the electrical output testing of the pPZT@Ag/PVDF PEHs. The voltage signals generated by the PEH present nearly identical amplitudes, with merely the direction being different, as depicted in Figure 8c,d. This observation affirms that the signals detected by the electrostatic meter are solely attributed to the piezoelectric effect of the PEH, effectively eliminating any influence from triboelectric effects or systematic errors. Figure 8e,f illustrate the voltage and current test results of the pPZT@Ag/PVDF PEH. The voltages and currents show a similar trend, increasing and then decreasing with increasing silver nanoparticle loading. This is due to the fact that the silver nanoparticles increase the local electric field strength, which promotes the polarization of pPZT and the formation of the *β*-phase. However, as silver nanoparticle loading increases, the PVDF produces unconfined structures and percolation paths, leading to a decrease in output. Notably, the *V*_oc_ and *I*_sc_ of pPZT@60Ag/PVDF PEH are the largest, 14.33 V and 0.93 μA, respectively, which are 2.56 and 2.82 times higher than those of pPZT/PVDF PEH. Based on these results, the pPZT@60Ag/PVDF PEH sample was selected for further comprehensive testing.

**Figure 9:**
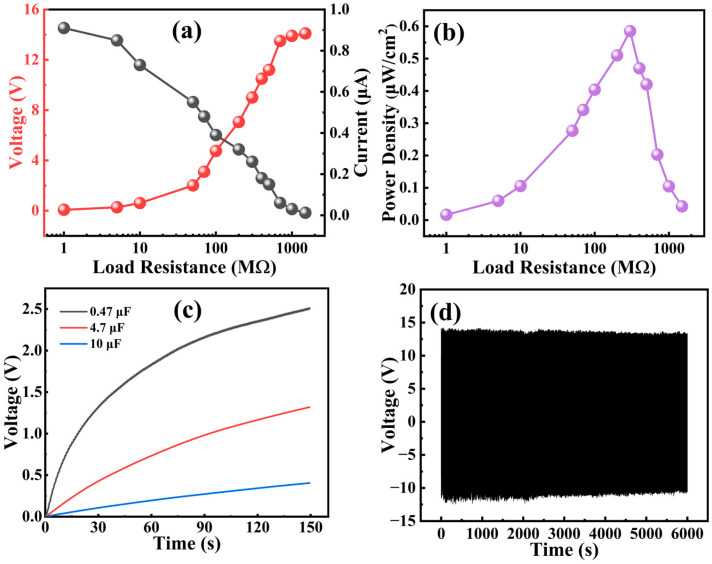
(**a**) Output voltage–output current and (**b**) power density of pPZT@60Ag/PVDF PEH under different loads. (**c**) Charging curves of pPZT@60Ag/PVDF PEH against different commercial capacitors and (**d**) after 6000-cycle endurance test.

We connected the pPZT@60Ag/PVDF PEH in both series and parallel arrangements with resistors of diverse resistance values (1 MΩ to 1.5 GΩ), as depicted in Figure S8a,b. As shown in Figure 9a, the voltage and current of the pPZT@60Ag/PVDF PEH show opposite trends to the load resistance. The power density of the pPZT@60Ag/PVDF PEH is calculated from Equation (5) [45].

(5) P=UI/S

where *U* and *I* represent the output voltage and current, and *S* represents the area of the electrodes (2 × 2 cm^2^). As shown in Figure 9b, the power density increases with the load resistance and decreases after reaching a peak of 0.58 μW/cm^2^ at 300 MΩ. Additionally, as shown in Table S1, the output voltage and power density of the pPZT@60Ag/PVDF PEH prepared by us are superior to those of recently published PEHs. Furthermore, the bridge rectifier can store the current generated by the PEH in a capacitor, as shown in Figure S8c. The charging process of the pPZT@60Ag/PVDF PEH for capacitors (0.47 μF, 4.7 μF, and 10 μF) is shown in Figure 9c. The voltage across each capacitor increases rapidly at the beginning of charging but slows down as time elapses. After charging for 150 s, the voltages across the 0.47 μF, 4.7 μF, and 10 μF capacitors are 2.51 V, 1.32 V, and 0.41 V, respectively. The durability and stability of PEHs are crucial for their practical applications. Therefore, we performed fatigue cycling tests on the pPZT@60Ag/PVDF PEH at a 4 mm amplitude and a 1 Hz bending frequency. As shown in Figure 9d, the output voltage of the PEH can still reach 95.8% of the initial value after 6000 cycles of testing with only a slight decrease, which indicates that the pPZT@60Ag/PVDF PEH has excellent fatigue resistance and outstanding stability.

**Figure 10:**
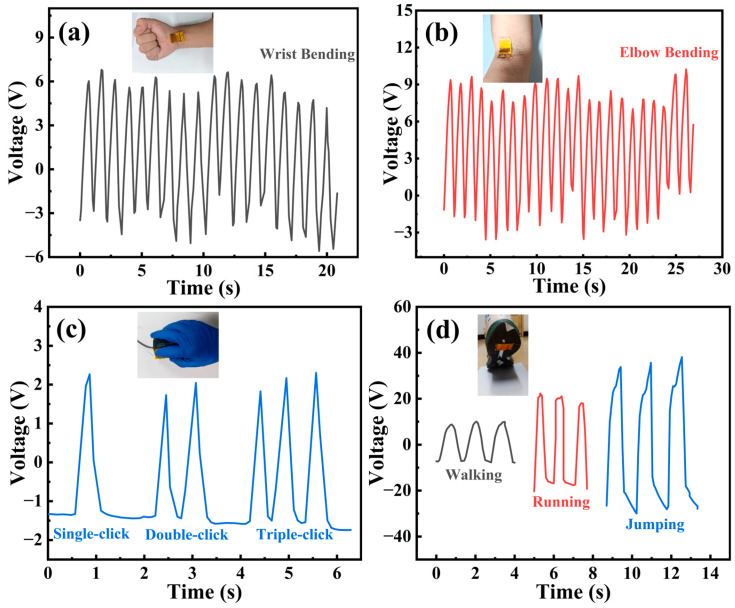
Electrical signals generated by pPZT@60Ag/PVDF PEH in response to different stimuli: (**a**) wrist flexion, (**b**) elbow flexion, (**c**) pressing mouse, and (**d**) heel pressure.

The development of PEHs has emphasized its application in the wearable field, especially for wearable energy harvesting under low-frequency (<5 Hz) human motion [46,47]. To confirm the possibility of using PEHs to convert mechanical energy generated by human movement, we tested the output response of the pPZT@60Ag/PVDF PEH under the stimulation of various human activities. As shown in Figure 10a,b, when placing the PEH on the wrist and elbow and bending the wrist and elbow back and forth, respectively, this behavior exerts a force of about 10 N and 30 N on the PEH, which can produce continuous and maximum voltage responses of 6.8 V and 10.2 V. Figure 10c shows that when placing the PEH on a mouse and clicking it continuously with different numbers of clicks, this process exerts a force of about 1.2 N on the PEH, which can respond to each knock. It can respond to each tap and generate a maximum voltage response of 2.3 V. When the PEH is fixed on the sole of a shoe and walking, running, and jumping actions are performed, as shown in Figure 10d, the above behaviors apply roughly 600 N, 900 N, and 1500 N of force to the PEH, respectively, and it can generate a maximum voltage response of 10.0 V, 22.3 V, and 38.2 V. The above experimental results confirm the possibility that the pPZT@60Ag/PVDF PEH can convert the mechanical energy generated by human movement into electrical energy and is expected to be used in pressure-sensitive insoles for gait rehabilitation monitoring.

## 4. Conclusions

In this study, silver nanoparticle-coated lead zirconate titanate fibers (pPZT@Ag) were fabricated through electrospinning coupled with a redox reaction, followed by integration into PVDF fiber matrices. This structural hybridization significantly enhanced the interfacial polarization efficiency and induced a 28.5% increase in the *β*-phase content (verified by FTIR), thereby optimizing the piezoelectric response. The pPZT@60Ag/PVDF composite fiber film exhibited a remarkable piezoelectric coefficient (*d*_33_ = 25 pC/N), representing a 122.5% enhancement over the unmodified counterpart, which is attributed to the synergistic effects of Ag-induced local field amplification and improved stress transfer efficiency. The corresponding piezoelectric energy harvester (PEH) demonstrated a peak open-circuit voltage of 14.33 V and a power density of 0.58 μW/cm^2^. The device maintains a voltage output retention after 6000 bending cycles (4 mm amplitude, 1 Hz), meeting durability requirements for wearable applications and promising pressure-sensitive insoles for gait rehabilitation monitoring in the future. Furthermore, further precise control of the silver–ammonia solution concentration gradient and reduction time is expected to promote a more uniform distribution of silver nanoparticles on the surface of PZT fibers, which is expected to amplify the polarized electric field and improve the output performance of the device.

## Data Availability

The original contributions presented in this study are included in the article; further inquiries can be directed to the corresponding authors.

## Supplementary Materials

The following supporting information can be downloaded at https://www.mdpi.com/article/10.3390/ma18071425/s1. Figure S1. Flow charts for the preparation of pPZT. Figure S2. The photographic images of the self-made pressure system used to measure the PEHs. Figure S3. XRD patterns of pPZT and pPZT@xAg. Figure S4. EDS spectra of pPZT@60Ag. Figure S5. The ratio of the content of the elements in pPZT@60Ag. Figure S6. Particle size distribution diagrams of (a) pPZT/PVDF composite fiber film and pPZT@Ag/PVDF composite fiber films with different silver nanoparticle loadings: (b) pPZT@20Ag, (c) pPZT@40Ag, (d) pPZT@60Ag, (e) pPZT@80Ag, (f) pPZT@100Ag. Figure S7. FoM of pPZT/PVDF and pPZT@Ag/PVDF composite fiber films. Figure S8. Test circuit diagrams: (a) parallel circuit, (b) series circuit, (c) rectifier circuit. Table S1. Comparison of piezoelectric output performance of the pPZT@60Ag/PVDF PEH with other PEHs reported previously. References [14,23,26,48,49,50,51,52] are cited in the Supplementary Materials.
