# Supplementary material for: Electrospun Silver-Modified PZT/PVDF Composites for High-Performance Piezoelectric Energy Harvester
Source: Materials (Basel). 2025 Mar 24;18(7):1425. doi: 10.3390/ma18071425 (PMC11989243; doi:10.3390/ma18071425)
Supplement: Supplementary file 1 [file materials-18-01425-s001.zip › materials-3485126-supplementary.pdf]

Supporting Information

Electrospun Silver-Modified PZT/PVDF Composites for High-performance Piezoelectric Energy Harvester

Xiang Liu [1, 2, 3], Huiling Guo [4], Xinyue Yang [1, 2], Fuling Wu [1, 2, 3], Yuanhui Li [1, 2], Xiao Li [5], Qing Xu [1, 2, *], Huajun Sun [1, 2, 3, *]

[1] State Key Laboratory of Silicate Materials for Architectures, Wuhan University of Technology, Wuhan 430070, China

[2] School of Materials Science and Engineering, Wuhan University of Technology, Wuhan 430070, Hubei, China

[3] Advanced Ceramics Institute of Zibo New & High-Tech Industrial Development Zone, Zibo 255000, Shandong, China

[4] College of Materials Science and Engineering, Hubei University of Automotive Technology, Shiyan 442002, China

[5] School of Chemistry, Chemical Engineering and Life Science, Wuhan University of Technology, Wuhan, 430070, China

*Corresponding author: huajunsun@whut.edu.cn (H. Sun) (TEL:13387590025),
xuqing@whut.edu.cn (Q. Xu) (TEL:13871033351)

The preparation process of dopamine-coated PZT nanofibers (pPZT) is shown in Figure S1. First, 5.1 mL of tetrabutyl titanate was added dropwise to a beaker containing 4.8 mL of acetylacetone and stirred for 30 minutes. Then 12 mL of anhydrous ethanol, 17 mL of glacial acetic acid, 7.925 g of zirconium acetylacetonate, and 8.940 g of alkaline lead acetate were added sequentially and stirred for 12 h to obtain the PZT precursor solution. PVP solution was added to the PZT precursor solution to adjust the viscosity, and then spun at a spinning electric field of 1.2 kV/cm, a collection drum speed of 1500 r/min, and a syringe flow rate of 1.5 mL/h. The PZT fiber membrane obtained from spinning was dried, calcined and milled to obtain PZT nanofibers (PZT NFs). 1 g of milled PZT NFs and 0.5 g of dopamine hydrochloride were added to 120 ml of Tris-HCl buffer and stirred for 14 h at 60°C in an oil bath. The suspension was centrifuged, washed, and dried to obtain pPZT.

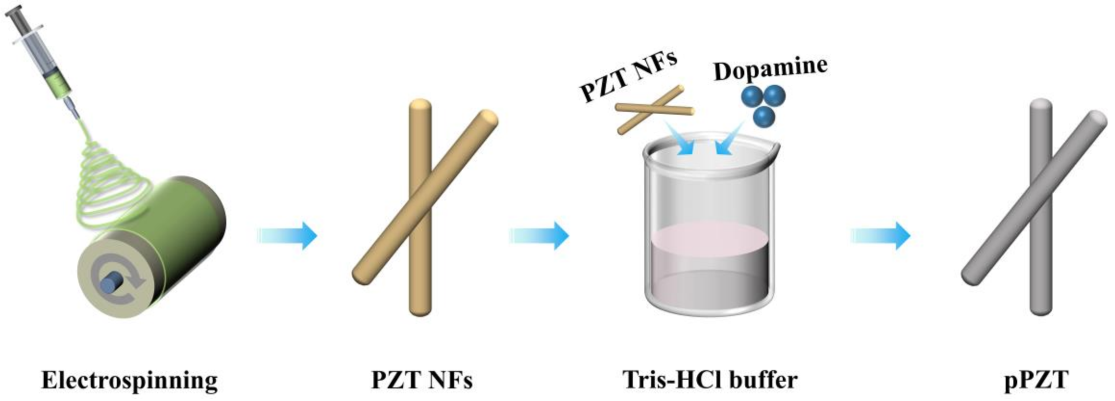

Figure S1. Flow charts for the preparation of pPZT.

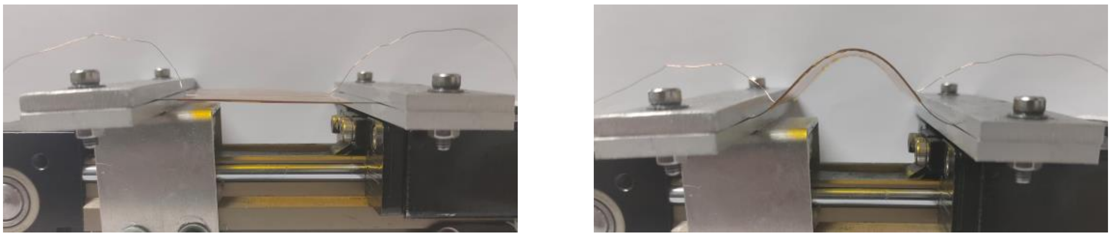

Figure S2. The photographic images of the self-made pressure system used to measure the PEHs.

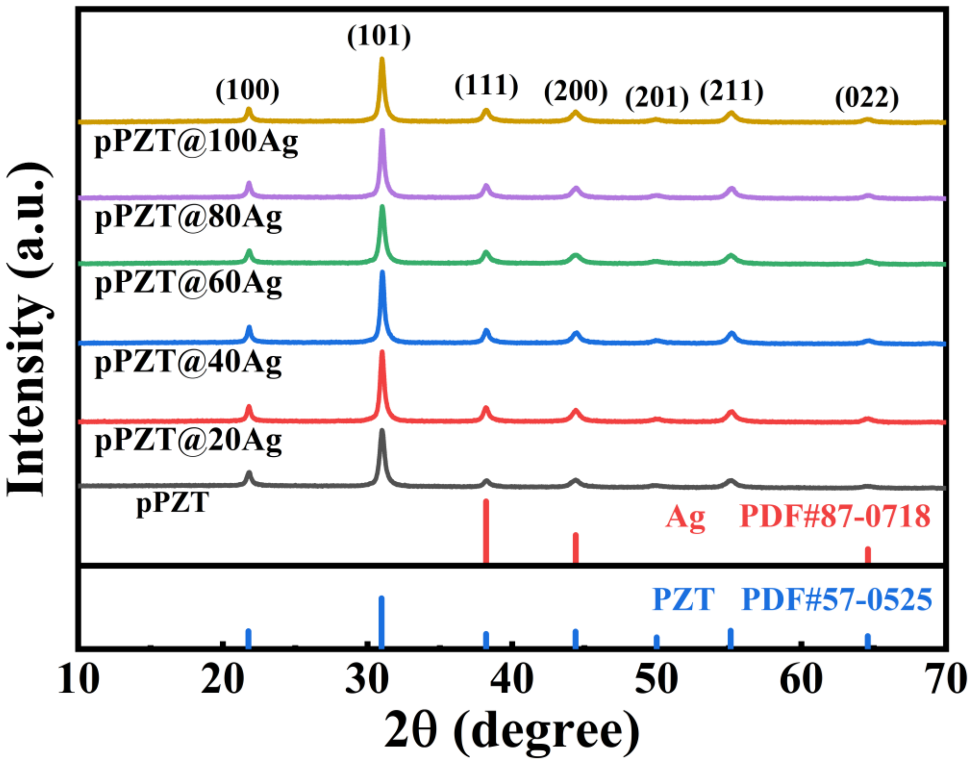

Figure S3. XRD patterns of pPZT and pPZT@xAg.

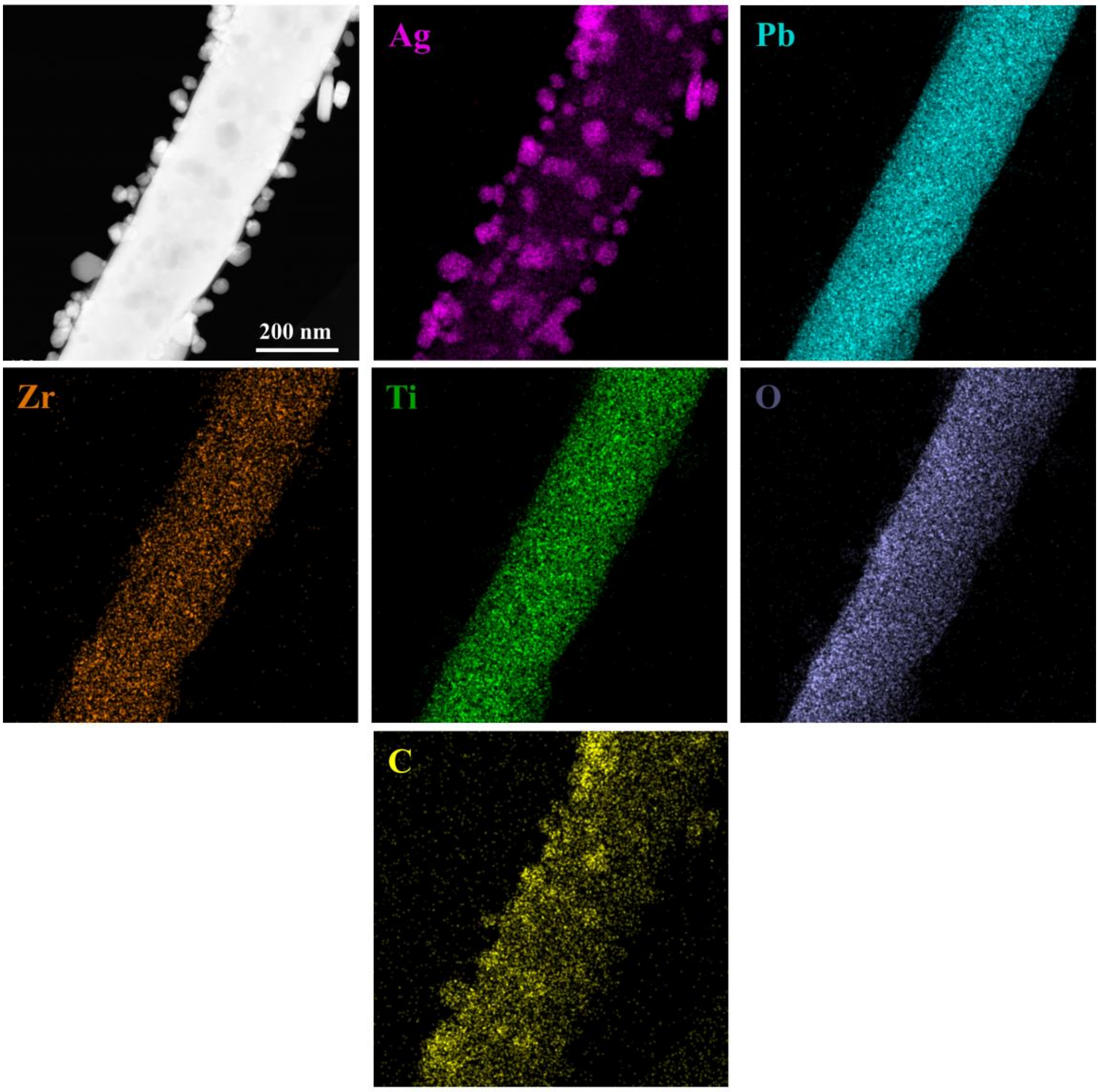

Figure S4. EDS spectra of pPZT@60Ag

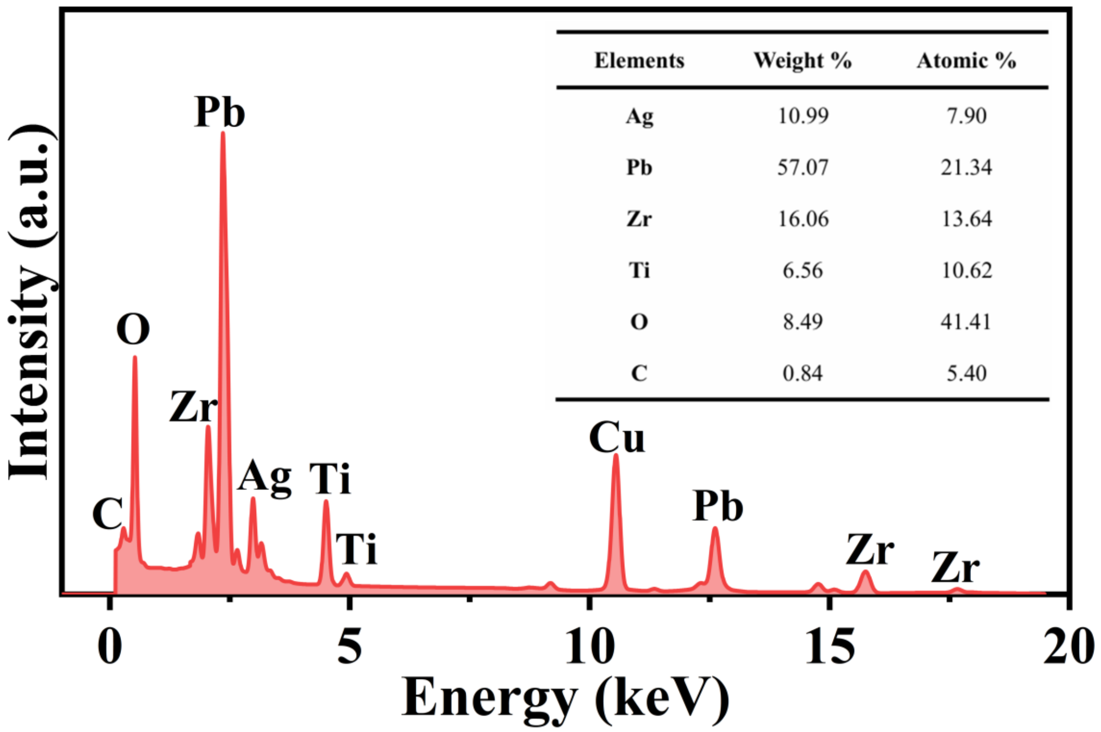

Figure S5. The ratio of the content of the elements in pPZT@60Ag.

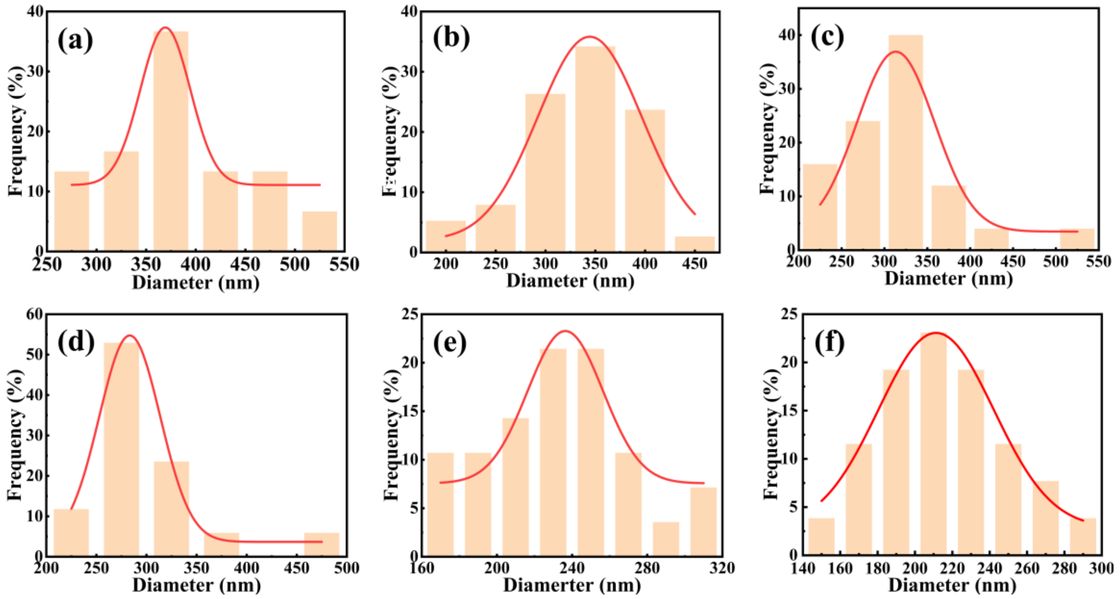

Figure S6. Particle size distribution diagrams of (a) pPZT/PVDF composite fiber film and pPZT@Ag/PVDF composite fiber films with different silver nanoparticle loadings: (b) pPZT@20Ag, (c) pPZT@40Ag, (d) pPZT@60Ag, (e) pPZT@80Ag, (f) pPZT@100Ag.

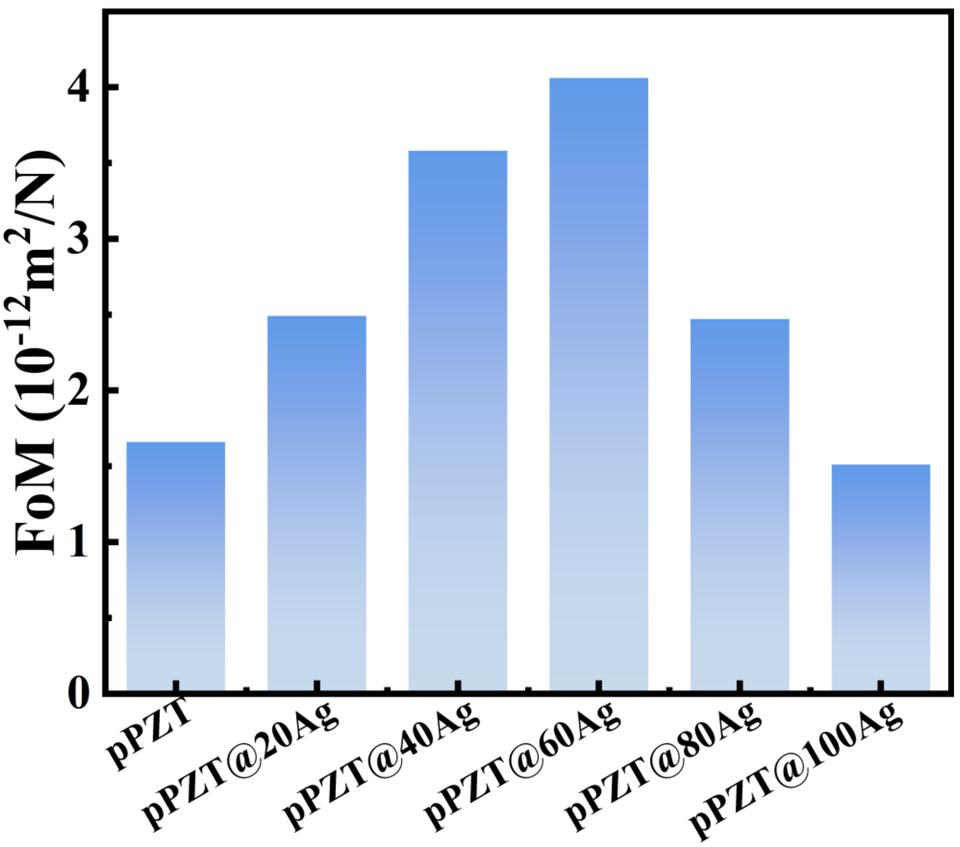

Figure S7. FoM of pPZT/PVDF and pPZT@Ag/PVDF composite fiber films.

Table S1. Comparison of piezoelectric output performance of the pPZT@60Ag/PVDF PEH with other PEHs reported previously.

Materials	Voltage (V)	Current (μA)	Power density (μW/cm^2)	Ref
PVDF/PZT/CNTs	9.15	1.14	-	[1]
PVDF/CNT	12	0.03	-	[2]
PVDF/PZT	10.2	0.86	0.54	[3]
PVDF/PZT	2.51	0.078	-	[4]
PVDF/$CaTiO_3$	20	0.25	0.19	[5]
F@BT/PVDF	2.1	0.49	0.60	[6]
PVDF/MXene	3.15	0.134	0.044	[7]
PVDF-HFP/BCZT	1.8	-	0.38	[8]
PVDF/pPZT@Ag	14.33	0.93	0.58	**This work**

[1] M. Liang, J. Wang, L. Su, et al., Versatile Lamellar Wrap-Structured PVDF/PZT/CNTs Piezoelectric Sensor for Road Traffic Information Sensing, Monitoring, and Energy Harvesting, Chemical Engineering Journal 497 (2024)

[2] S. Badatya, D.K. Bharti, N. Sathish, et al., Humidity Sustainable Hydrophobic Poly(vinylidene fluoride)-Carbon Nanotubes Foam Based Piezoelectric Nanogenerator, Acs Applied Materials & Interfaces 13 (23) (2021) 27245-27254.

[3] X. Li, C. Yuan, H. Zhou, et al., A novel PZT hollow structure utilized in high-performance piezoelectric nanogenerator, Ceramics International 50 (19) (2024) 34756-34767

[4] G. Tian, W. Deng, Y. Gao, et al., Rich lamellar crystal baklava-structured PZT/PVDF piezoelectric sensor toward individual table tennis training, Nano Energy 59 (2019) 574-581.

[5] S. Panda, S. Hajra, H. Jeong, et al., Biocompatible CaTiO3-PVDF composite-based piezoelectric nanogenerator for exercise evaluation and energy harvesting, Nano Energy 102 (2022)

[6] S. Wang, Z. Yu, L. Wang, et al., A core-shell structured barium titanate nanoparticles

for the enhanced piezoelectric performance of wearable nanogenerator, Applied Energy 351 (2023)

[7] J. Zhang, T. Yang, G. Tian, et al., Spatially Confined MXene/PVDF Nanofiber Piezoelectric Electronics, Advanced Fiber Materials 6 (1) (2023) 133-144.

[8] K. Jeder, A. Bouhamed, H. Nouri, et al., Enhancement of the performance of flexible lead-free nanogenerators by doping in BaTiO3 nanoparticles, Energy 261 (2022)

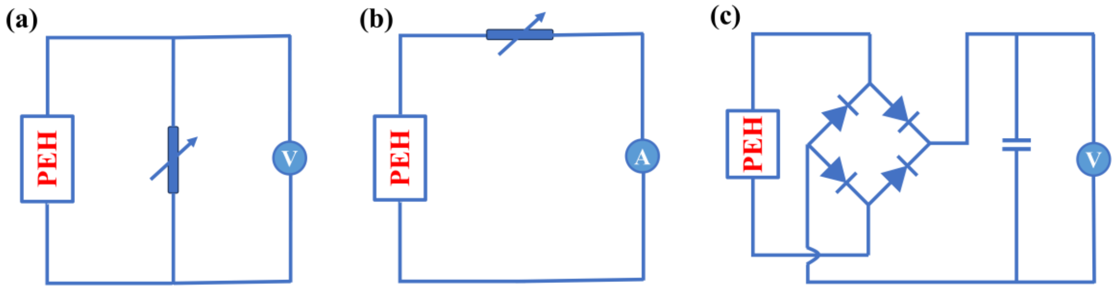

Figure S8. Test circuit diagrams: (a) parallel circuit, (b) series circuit, (c) rectifier circuit.
